# Indoor Air Quality and Respiratory Health among Malay Preschool Children in Selangor

**DOI:** 10.1155/2015/248178

**Published:** 2015-04-23

**Authors:** Nur Azwani Mohd Nor Rawi, Juliana Jalaludin, Poh Choo Chua

**Affiliations:** Department of Environmental and Occupational Health, Faculty of Medicine and Health Sciences, Universiti Putra Malaysia, 43400 Serdang, Selangor, Malaysia

## Abstract

Indoor air quality (IAQ) has been the object of several studies due to its adverse health effects on children. *Methods*. A cross-sectional comparative study was carried out among Malay children in Balakong (2 studied preschools) and Bangi (2 comparative preschools), Selangor, with the aims of determining IAQ and its association with respiratory health. 61 and 50 children aged 5-6 years were selected as studied and comparative groups. A questionnaire was used to obtain an exposure history and respiratory symptoms. Lung function test was carried out. IAQ parameters obtained include indoor concentration of particulate matter (PM), volatile organic compounds (VOCs), carbon monoxide (CO), carbon dioxide (CO_2_), temperature, air velocity (AV), and relative humidity. *Results*. There was a significant difference between IAQ in studied and comparative preschools for all parameters measured (*P* < 0.001) except for CO_2_ and AV. Studied preschools had higher PM and CO concentration. FVC, FEV_1_, FVC% and FEV_1_% predicted values were significantly lower among studied group. Exposures to PM, VOCs, and CO were associated with wheezing. *Conclusion*. The finding concluded that exposures to poor IAQ might increase the risk of getting lung function abnormality and respiratory problems among study respondents.

## 1. Introduction

Indoor air quality (IAQ) has been the object of several studies due to an increasing concern within the scientific community on the effects of IAQ upon health, especially when people tend to spend more time indoors than outdoors [1]. Frequently, pollutants from indoor sources may build up to appreciable levels due to the slowness of air exchange. It is estimated that a quarter of the world population is exposed to unhealthy concentrations of air pollutants and children are the ones most at risk of these indoor air pollutants due to their respiratory organ systems immaturity [2]. Moreover, children are susceptible to air pollutants because they breathe in relatively greater air volume than adults.

The aim of this study is to determine the IAQ and its association with respiratory health among Malay preschool children in Selangor. Preschool children are particularly advantageous when studying respiratory symptoms and air pollution because they are unlikely to smoke cigarettes regularly, have no serious exposure to occupational pollutants, and tend to have a stable residential history, and their respiratory system seems to be more sensitive to air pollution. There were very limited numbers of studies that relate IAQ to the health impacts in Malaysia. This research will help to increase awareness to the community, especially the parents and teachers on the risk of poor IAQ.

## 2. Methods

### 2.1. Study Location/Study Design

This cross-sectional study was carried out among male and female Malay children who attended preschools in Balakong area (studied group), while the population for comparative group was selected among male and female Malay children who attended preschools in Bangi area.

### 2.2. Study Population/Sampling Frame/Sample Size/Sampling Technique

A total of 111 preschool children aged 5-6 years were recruited from 4 preschools located in Balakong and Bangi, Selangor. Studied populations were selected among male and female Malay children who attended preschool in Balakong area, while the population for comparative group was selected among male and female Malay children who attended preschools in Bangi area. Random sampling method was used to select the respondents with several inclusion criteria; only preschool children ranging from 5 to 6 years old, healthy, Malays, and free from any respiratory illness were selected. The name list of the children was obtained from the preschool teachers. Screening of respiratory illnesses was done by using a questionnaire and those who reported respiratory illnesses were excluded from the study. Children's parents were responsible for answering the questionnaires and for passing back to the school teacher, which was then being collected by the researcher.

### 2.3. Instruments and Procedures

The questionnaires used were adopted from The American Thoracic Society, “Questionnaire ATS-DLD-C WHO (1982).” The questionnaire was pretested and the total respondents for the pretest were 10% of the sample size. Indoor air quality assessments were conducted in each preschool using several indoor air monitoring instruments. The monitoring phase included air sampling for at least a 3- to 4-hour period during preschool normal activities. The IAQ monitoring instruments used in this study included TSI 8520 DustTrak Airborne Particle Monitor for PM_2.5_ and PM_10_; PbbRAE Portable VOC Monitor (pbbRAE 3000) for VOCs; Q-Trak Plus Model 8554 Monitor for CO_2_, CO, relative humidity, and temperature; and TSI Velocicalc Plus Model 8386 for air velocity. Instruments for PM_2.5_, PM_10_, and VOCs were placed at a height of about 0.6–1.5 meters above the floor, approximately at the level of children's breathing zone. The selected place was not closer than 1 meter to a wall, a door, or an active heating system. Whenever possible, all the instruments were placed at the back of the classroom to avoid any disruption of sound from the instruments during a learning session and to avoid attraction for the children. Meanwhile, the measurements of CO_2_, CO, temperature, relative humidity, and air velocity were taken periodically and spread throughout many areas in the building to be sure that they were distributed evenly. MM-SP004 Tabletop Portable Spirometer was used to conduct a lung function test among the study respondents. In this study, evaluation of lung function test was performed by comparing the obtained value with normal values (standard value). Based on a study by Azizi and Henry [3], predicted value was calculated.

### 2.4. Data Analysis

Statistical analysis was carried out using SPSS version 20.0. To study the association and differences between indoor air pollutant concentrations and the respiratory health of children, *t*-test, Mann-Whitney *U* test, and Chi-square test were used. The multiple regression test was performed to determine the main variables that influence the respiratory health of the children.

## 3. Results

### 3.1. Sociodemography Data of Respondents

According to the results obtained, the majority of fathers in the studied group have an education level of degree or Ph.D. degree, while the majority of fathers in the comparison groups only studied until SPM level. As for mother's education level, the majority of mothers in both groups studied until end of Form 5 or STPM level. The Chi-square test showed that there were significant differences between the parental education level between studied and comparative groups. As for the total household income, the mean income for the studied group was RM (4369.79 ± 2747.31) and RM (3213.98 ± 1687.87) for the comparative group. The comparison between total dwellers for both studied groups was also done where the mean for total dwellers in the house for studied group was (5.43 ± 1.36) and (5.72 ± 1.84) for comparative group. Total number of rooms available in the house were also compared where the mean of total room in studied and comparative groups was (3.16 ± 0.66) and (3.52 ± 1.11), respectively. Results from Mann-Whitney *U* test shows that there were significant differences in terms of total household income (*Z* = −3.390, *P* < 0.001) between the study groups, whereas there was no significant difference found between the years living in the vicinity between the study groups.

### 3.2. Comparison of Preschools Building Characteristics

Data samples from these 4 preschools have been compared. The preliminary site survey showed that more than all of the preschool buildings were aged below 10 years. Most of the preschool building walls were made up of the concrete and cemented floor. All the preschools were naturally ventilated where they used fan as their mechanical devices for ventilation purpose. All the preschools have more than 4 windows in the preschools' building. Preschools from studied area cleaned their preschool twice daily. All 2 studied group preschools have cooking activities in their premises. All preschools involved in this study were using insulation board for the preschools' building ceiling. Preschools from the comparative group were using book shelf made up of pressed wood. All these preschools cleaned their floor once a day. Preschools from studied group reported the highest number of heavy traffic density as compared to comparative group preschools. All the preschools reported most of the outdoor pollution was originated from vehicles.

### 3.3. Comparisons of Indoor Air Quality between Study Areas

Table 1 shows that the mean of indoor PM_2.5_ in studied area was higher as compared to comparative area. Mann-Whitney *U* test found a significant association between the levels of indoor PM_2.5_ in both studied areas (*Z* = −5.494, *P* < 0.001). The mean indoor concentration of PM_10_ was higher in the studied area compared to the comparative area. Statistical analysis also showed a significant difference in indoor PM_10_ levels between studied and comparative areas (*Z* = −6.445, *P* < 0.001). The mean of volatile organic compounds (VOCs) in classrooms was found higher in comparative area compared to studied area. Results show that there was a statistical significant difference between indoor VOCs levels in studied and comparative areas (*Z* = −2.214, *P* < 0.05).

Parametric test revealed that the distribution of CO_2_ in classrooms for studied and comparative areas was not normal, with the mean of CO_2_ in the comparative area being higher than the studied area. The distribution of CO_2_ between studied and comparative areas indicates no significant difference, whereas, for CO, the mean was higher in the studied area as compared to comparative area. There was a statistical significant difference between CO levels in classrooms in studying and comparative areas (*Z* = 7.866, *P* < 0.001). As for the physical parameters, there were significant differences between temperature (°C) and relative humidity (%) in both areas with (*Z* = −7.143, *P* < 0.001) and (*Z* = −2.821, *P* < 0.05).

### 3.4. Comparison of Lung Function

Results from statistical analysis showed that the lung function among comparative group was significantly higher as compared to studied group. Findings from the analysis show that all lung function parameters (FVC, FEV_1_, FVC%, and FEV_1_%) were significantly higher for the comparative group as compared to the studied group except for FEV1/FVC% (Table 2).

### 3.5. Prevalence of Respiratory Symptoms

Four parameters of respiratory symptoms were assessed in this study, where the symptoms were identified using the standardized and validated questionnaires, adopted from American Thoracic Society “Questionnaire ATS-DLD-C WHO” (1982). Questions asked included cough, phlegm, wheezing, and chest tightness experienced by children.  Table 3 shows that most of the children who reported for cough (21 of them) were from the studied group (34.4%) as compared to 7 (4.0%) of them from the comparative group. Other symptoms such as phlegm did not commonly occur among children involved in this study as reported by parents, only 3 (4.9%) children from studied group and 1 (2.0%) child from comparative group. Wheezing was another common symptom found among children attending preschool in studied group (20 (32.8%)) but not for children in comparative group (8 (16.0%)). Very few reports of chest tightness were experienced among children with only 1 (1.6%) in studied children and none in comparative children.

### 3.6. Association between Indoor Air Pollutants and Lung Function among Study Respondents

The indoor air pollutant concentrations were categorized based on median value. A value that was higher than median was categorized as high while the value that was lower than median was categorized as low. The results showed there was a significant association between indoor concentration of PM_2.5 _and abnormality of FVC% among study respondents (*P* = 0.014). Significant association was also found in the concentration of CO with the abnormality of FVC% (*P* = 0.003). However, there was no association found between PM_10_, VOCs, and CO_2_ with the abnormality of FVC% among study respondents. Similarly, there were no associations found between indoor air pollutants and the abnormality of FEV_1_%, except for CO (*P* = 0.003).

### 3.7. Association between Indoor Air Pollutants and Respiratory Symptoms

The associations between indoor PM_2.5_, PM_10_, VOCs, CO_2_, and CO inside different classrooms in preschools with the prevalence of respiratory symptoms were established using the median value. Results from statistical analysis showed a significant association between wheezing and indoor PM_2.5_ concentrations in preschools (OR = 2.69, 95% CI = 1.07–6.79) whereas no significant associations were found between cough, phlegm and chest tightness with indoor PM_2.5_ concentration. The median value was also used to categorize indoor PM_10_ concentration in preschools. Chi-square test reveals that there was a significant association between wheezing and indoor PM_10_ concentrations (OR = 5.31, 95% CI = 1.70–16.68), while no significant associations found for other respiratory symptoms.

Indoor VOCs concentration was categorized into high (≥0.103 ppm) and low (<0.103 ppm) values. From the results obtained, there were no significant associations found between phlegm and chest tightness with indoor VOCs concentrations. However, there were significant associations found between cough and wheezing with indoor VOCs concentration (OR = 3.62, 95% CI = 1.29–8.25) (OR = 0.23, 95% CI = 0.09–0.61). High and low values for both indoor CO_2_ and CO concentrations were also categorized using median values. There was no significant association found between indoor CO_2_ concentration and respiratory symptoms. Indoor CO concentrations also showed no significant association with respiratory symptoms, except for wheezing (OR = 5.78, 95% CI = 1.62–20.70).

### 3.8. Factor Influenced the Abnormality of Lung Function among Study Respondents after Controlling All the Confounders

Logistic regression was conducted to determine the main factor that influenced the abnormality of FVC% and FEV_1_% among study respondents after controlling all the confounders in this study.  Table 4 showed that the abnormality of FVC% among children was significantly associated with the concentrations of PM_2.5_ and CO.  Table 5 showed that the abnormality of FEV_1_% among children was significantly associated with the concentration of CO after controlling all the confounders.

### 3.9. Factor Influenced Wheezing among Study Respondents after Controlling All the Confounders

Logistic regression was carried out in order to determine the factors that influenced wheezing symptoms among study respondents after controlling all the confounders such as income, parental education level, indoor smoking, and duration living in the vicinity.  Table 6 showed that wheezing had significant association with the concentrations of PM_2.5_, PM_10_, and CO.

## 4. Discussion

### 4.1. Sociodemography Data of Respondents

111 Malay preschool children between 5 and 6 years old participated in this study. This study was carried out at 4 different preschools; 2 preschools from Balakong (studied group) and 2 preschools from Bangi (comparative group) were selected. Both studied areas consist of 8 classrooms, with 4 classrooms for each area. Classrooms A, B, C, and D were located in the studied area; Classrooms E, F, G, and H were located in the comparative area. Respondents were categorized into two sample units, studied and comparative groups. Sociodemographic factors were successfully matched as obtained in the statistical analysis where it discovered no significance differences in terms of total dwellers in both studied and comparative areas. The years living in the vicinity among study groups were also assessed as to consider their residential exposures. The majority of studied children live in the urban area while the majority of the comparative children live in a suburban area. Most children who live in urban area live in close proximity to the main road compared to those who live in a suburban area.

### 4.2. Comparisons of Indoor Air Quality between Study Areas

Indoor air quality parameters were measured from 8 different classrooms in this study. The highest concentrations of PM_2.5_ (94 *μ*g/m^3^) and PM_10_ (131 *μ*g/m^3^) were measured in Classroom A, which was located in the studied location. Accordingly, the highest VOCs (0.12 ppm) were found in Classroom F, and the highest CO_2_ (1073 ppm) was found in Classroom C. Classroom D showed the highest CO with the concentration of 2.60 ppm. Temperature, relative humidity, and air velocity were recorded higher within preschools in the studied area, as compared to preschools in the comparative area. In order to feel comfortable, Department of Occupational Safety and Health (DOSH) stated that the indoor temperature should be in between 23 and 26 degrees Celsius (°C). Too little humidity in a space may create static build up and people will sense that their skin feels dry, whereas too much humidity will cause skin to feel sticky. According to American Society of Heating, Refrigerating, and Air Conditioning Engineers (ASHRAE) Standard 55, indoor humidity levels should be monitored between 30 and 65% for optimum comfort level. The acceptable range of air movement was between 0.15 and 0.50 m/s. Inadequate ventilation, high temperature, and humidity can increase concentrations of some indoor pollutants.

Statistical analysis indicated that there was a significant difference in the concentration levels of PM_2.5_ and PM_10_ between studied and comparative areas and suggested that the preschool locations might have contributed to the concentration of the particulates. The location of preschools as well as outdoor and indoor combustion activities is the major contributor to the high level of indoor PM_2.5_ and PM_10_ in the studied area. Both preschools in the studied area had a kitchen in the building. Cooking activity inside the building might have contributed to higher PM_2.5_, compared to preschools in the comparative area. Cooking indoor can also generate particles <0.1 *μ*m, which accounted for 30% of the particle volume as shown by Kamens et al. [4]. Motor vehicles are the major contributors to particles in urban air pollution, emitting fine primary particles.

Outdoor sources of particulate matters such as generation of dust from paved or unpaved roads might also contribute to the high level of indoor particulate matter. Differing from preschools in comparative area, both preschools in the studied area were located nearby busy roads. Thus, heavy traffic and vehicle fossil fuel combustion might have contributed to high levels of particulate matters in studied areas. Rom [5] reported that heavy traffic and highly polluted area, especially during early in the morning and afternoon, would fabricate more PM_10_. Moreover, indoor PM_10_ concentrations appear to be high in buildings located in urban areas or close to motorways [6]. In this study, the urban preschool was using the open ventilation system, where outdoor air pollutants might easily go into the classrooms.

Statistical analysis showed that there was a significant difference between indoor VOCs levels between the studied and comparative areas. The indoor concentrations of VOCs usually exceeded outdoor levels [7]. VOCs concentration levels were slightly higher in comparative area compared to the studied area due to the presence of VOCs-emitting materials in preschools buildings. Both of the preschools in the comparative area used wood for the school furniture, including children's desks and chairs. Most of them were made of pressed woods, where these pressed wood products sometimes contain urea-formaldehyde, which may have released gas formaldehyde over a substantial period of time. According to EPA [8], the rate at which products like pressed wood release formaldehyde can change. Formaldehyde emissions will generally decrease as products age. When the products are new, high indoor temperatures or humidity can cause increased release of formaldehyde from these products. Classroom F which was located in the comparative area registered for the highest concentrations of VOCs (0.12 ppm). Moreover, this preschool closed all the doors and windows during lessons as to keep children inside, resulting in poor ventilation. The inadequate ventilation may favor the accumulation of pollutants inside, combined with other additional indoor sources. Wålinder et al. [9] found that the indoor concentrations of VOCs were the highest (2–8 times higher) in the school that had the lowest ventilation rate.

A significant difference was also found in the mean concentrations of CO_2_ and CO in the studied area. The highest concentration of CO_2 _was found in Classroom C, which was located in the studied area. The doors and windows were closed during each lesson, which contributed to less air exchange. The total number of persons in this classroom can be up to more than 20 persons each time, causing overcrowding. A study in Portugal reveals a strong correlation between the CO_2_ levels with occupancy [10]. During the work period in the city center school, the CO_2 _levels ranged widely from 899 to 2540 mg/m^3^, while, in the suburban school, the values were between 833 and 1859 mg/m^3^. In this study, the highest concentration of CO (2.6 ppm) was found in Classroom D, which was located in the studied area. A higher concentration of CO was majorly contributed by mobile vehicles, as well as the close proximity of the preschool itself with the busy roads, especially during peak hours. Therefore, the children might be exposed to the traffic air pollutants that went inside their classrooms. This usually occurred in the morning, when the school buses and parents congested the preschool areas.

### 4.3. Comparison of Lung Function

*t*-test analysis was performed to compare the values of the FVC (liter), FEV_1 _(liter), FVC%, and FEV_1_% among studied and comparative children. The test reveals that FVC (liter), FEV_1 _(liter), FVC%, and FEV_1_% values were significantly higher among the comparative children compared to the studied children (*t* = −4.160, *P* < 0.001), (*t* = −4.484, *P* < 0.001), (*t* = −4.811, *P* < 0.001), and (*t* = −4.577, *P* < 0.001). Meanwhile, Mann-Whitney *U* Test was used to compare the values of FEV_1_/FVC% among studied and comparative children where statistical analysis reveals that the values of FEV_1_/FVC% among studied and comparative children were not significantly different. The median for FEV_1_/FVC% was 103.69 ± 05.67 and 105.62 ± 6.81 for both studied and comparative groups.

A study conducted in Klang Valley by Fariza et al. [11] found an association between PM_2.5_, PM_10_, and VOCs with lung function test (FEV_1_ and FVC) among school children of urban area, where the lung function among children in urban was significantly lower than those who live in rural area. According to study done by Fritz and Herbarth [12], it shows that the pulmonary functions of the preschool children who lived in urban areas, especially male children, were very sensitive to the deleterious effects of the high ambient pollution levels as compared to the comparative group children. Besides, exposures to a pollution profile of heavy traffic also showed markedly lower FVC and FEV_1 _among preschool children in Leipzig, Germany.

### 4.4. Prevalence of Respiratory Symptoms

Results from Chi-square test reveal that the prevalence of respiratory symptoms was higher among studied children for cough (OR = 3.23, 95% CI = 1.24–8.40) and wheezing (OR = 2.56, 95% CI = 1.02–6.47). In Malaysia, a number of studies conducted locally showed that air pollutants can worsen childhood asthma even at low concentrations [13, 14]. The study conducted by Zakaria et. al, [15] among a total of 207 school children in Klang Valley revealed that the presence of particulate matter can influence the severity of asthma among primary school children in urban, industrial, and rural areas of Selangor and Kuala Lumpur. Study by Pierse et al. [16] reported a higher prevalence of cough without cold in a cohort of 4400 preschool children with increased exposure to locally generated particulate matter pollution mainly from the road. According to a study by Jerrett et al. [17], asthmatic children in Taiwan who were exposed to high levels of traffic related air pollution also reported more respiratory symptoms than children with lower exposures. Associations between VOCs exposures and poor respiratory health were also observed in preschool children in a study conducted by Rumchev et al. [18]. Overall, the prevalence of respiratory symptoms was higher among children from studied group.

### 4.5. Association between Indoor Air Pollutants and Lung Function among Study Respondents

Significant associations were found between indoor concentrations of PM_2.5_ and CO with the abnormality of FVC%, while only concentrations of CO were found significant with the abnormality of FEV_1_%. This finding was supported by a study conducted in Klang Valley, where significant associations were found between indoor concentration level PM_2.5_ and the decrements of lung function among children in urban area [11]. There was also significant decrement found in lung function in a long-term study of air pollution in school-aged children in southern California [19]. This study found that decrements in lung function were related to exposures to PM_2.5_ and it demonstrated that air pollution affects the growth of lung function during the period of rapid lung development between the ages of 10 and 18 years. A population-based epidemiologic study in Taiwanese communities was published to show that traffic-related pollutants, CO, had chronic harmful effects on lung function of children [20].

### 4.6. Association between Indoor Air Pollutants and Respiratory Symptoms

Statistical analysis was carried out to assess the association between indoor air pollutant concentrations inside classrooms with respiratory symptoms among children. It is reported that there were significant associations found between PM_2.5_, PM_10_, VOCs, and CO with respiratory symptoms but not for CO_2_. A significant association between wheezing with indoor PM_2.5_ and indoor PM_10_ concentrations in preschools was found in this study. The long-term effects of PM_2.5_ particles on children include both lung function changes and the development of chronic respiratory disease, while short-term exposures to PM_10_ can result in increased respiratory symptoms. The concentrations of PM_10_ were associated with wheezing among asthmatic school children in a study conducted by Zakaria et al. [15].

Similarly, study by Sonnenschein-van der Voort et al. [21] suggests that long-term exposure to higher levels of traffic-related air pollutants such as PM_10_ is associated with increased risk of wheezing in the first 3 years of life. Associations of PM_2.5_ with overall wheezing until the age of 8 years were observed by Gehring et al. [22] in another study in The Netherlands. There were significant associations found between cough and wheezing with indoor VOCs concentration. This finding was consistent with the findings by Roy et al. [23]. The study states that the acute effects of VOCs are often associated with building related illness (BRI) syndrome. Gauderman et al. [19] found that domestic exposure to VOCs or household chemical products can increase the risk of asthma-like wheeze in preschool children. Low levels of CO can also cause health effects and trigger asthma. Accordingly, this study showed that indoor CO concentrations showed no significant association with respiratory symptoms, except for wheezing.

### 4.7. Factor Influenced the Abnormality of Lung Function among Study Respondents after Controlling All the Confounders

Logistic regression was conducted to determine the main factor that influenced the abnormality of FVC% and FEV_1_% among study respondents after controlling all the confounders in this study. Statistical analysis results reveal that the abnormality of FVC% among children was significantly associated with the concentrations of PM_2.5_ and CO. Short-term PM_2.5_ exposures are linked to reduced lung function, especially in children. An increase of 10 *μ*g/m of PM_2.5_ was associated with decreases of 3.5 mL FVC (95% confidence interval = −4.3 to −2.7) and 1.5 mL/year FVC growth (−2.0 to −1.0) in a study conducted by Roy et al. [23] among Chinese children.

A study in southern California by Gauderman et al. [19] over an eight-year period found that deficits in the growth of FEV_1 _were associated with exposure to elemental carbon (*P* = 0.007), even after adjustment for several potential confounders and effect modifiers. Most of the children in studied groups live less than 100 meters from the main road, whereas most of the children in comparative group live within 500–1000 meters from the main road. Living close to a street with high traffic was a predictor of personal exposure to particulate matters in children. Recent study by Gauderman et al. [19] also emphasized the importance of proximity to freeways as another factor affecting lung function in children.

### 4.8. Factor Influenced Wheezing among Study Respondents after Controlling All the Confounders

Logistic regression was carried out in order to determine the factors that influenced wheezing symptoms among study respondents after controlling all the confounders such as income, parental education level, indoor smoking, and duration living in the vicinity. Statistical analysis results reveal that wheezing had significant association with the concentrations of PM_2.5_, PM_10_, and CO. Wheezing is one of the classic symptoms associated with asthma in children. Short-term PM_2.5_ exposures were linked to increased hospital admissions and emergency department visits for respiratory effects, such as asthma attacks, as well as increased respiratory symptoms, such as wheezing. Research based on parental reports of symptoms also showed elevated rates of wheeze in association with PM_2.5_ and PM_10_ among preschoolers [16]. A study conducted by Zakaria et al. [15] also revealed that the presence of particulate matter, which is the PM_10_, influenced the severity of asthma among primary school children in urban, industrial, and rural areas of Selangor and Kuala Lumpur.

## 5. Conclusion

Findings from this study indicated that the exposures to poor indoor air quality and increasing levels of indoor pollutant concentrations were the risk factors that had caused a reduction in lung function and increasing reports of respiratory symptoms among the study respondents. This study suggests that knowledge should be given to the public, preschool managements, and parents, specifically about the risk of getting respiratory problems due to poor indoor air quality. Further studies are needed to confirm the observed association between indoor air pollutant concentrations and respiratory health among preschool children in urban, suburban, and rural areas.

## Figures and Tables

**Table 1:** Comparisons of indoor air quality between study areas.

Variables	Studied area (*n* = 61)	Comparative area (*n* = 50)	*Z*	*P*
	Median (IQR)		
PM_2.5_ (*µ*g/m^3^)	80.0 (17.0)	72.0 (19.0)	−5.494	**<0.001** ^*^
PM_10_ (*µ*g/m^3^)	118.0 (46.5)	54.0 (42.0)	−6.445	**<0.001** ^**^
VOCs (ppm)	0.08 (0.03)	0.11 (0.05)	−2.214	**0.027** ^*^
CO_2_ (ppm)	579.0 (340.0)	784.0 (526)	−0.287	0.774
CO (ppm)	1.0 (0.4)	0.8 (1.0)	−8.076	**<0.001** ^**^
Temperature (°C)	29.7 (3.5)	26.8 (2.3)	−7.143	**<0.001** ^**^
Rh (%)	78.1 (13.5)	54.7 (31.7)	−2.821	**0.005** ^*^
Air velocity (m/s)	1.59 (2.34)	0.97 (0.24)	−1.046	0.295

Mann-Whitney *U* Test.

^**^Significant at *P* < 0.001.

^*^Significance at *P* < 0.05.

*N* = 111.

**Table 2:** Comparisons of lung functions among preschool children.

Lung function	Studied group (*n* = 61)	Comparative group (*n* = 50)	*Z*/*t* value	*P* value
	Mean ± S.D/median ± IQR		
FVC (Liter)^a^	0.63 ± 0.18	0.79 ± 0.21	−4.160	**<0.001** ^*^
FEV_1_ (Liter)^a^	0.60 ± 0.17	0.76 ± 0.19	−4.484	**<0.001** ^*^
FVC%^a^	69.93 ± 17.03	85.94 ± 19.81	−4.577	**<0.001** ^*^
FEV_1_%^a^	72.02 ± 18.17	88.69 ± 18.16	−4.811	**<0.001** ^*^
FEV_1_/FVC%^b^	103.69 ± 05.67	105.62 ± 6.81	−1.671	0.095

^∗^Significant at *P* < 0.001.

^a^ *t*-test.

^b^Mann-Whitney *U* Test.

**Table 3:** Prevalence of respiratory symptoms among studied and comparative preschool children.

Variables	Studied (*n* = 61)	Comparative (*n* = 50)	*χ* ^2^ value	*P* value	OR^b^	95% CI
	Total (%)				
Cough						
Yes	21 (34.4)	7 (14.0)	**6.078**	**0.014** ^*^	**3.23**	**1.24**–**8.40**
No	40 (65.6)	43 (86.0)				
Phlegm						
Yes	3 (4.9)	1 (2.0)	0.674	0.412	2.53	0.26–25.15
No	58 (95.1)	49 (98.0)				
Wheezing						
Yes	20 (32.8)	8 (16.0)	**4.105**	**0.043** ^*^	**2.56**	**1.02**–**6.47**
No	41 (67.2)	42 (84.0)				
Chest tightness						
Yes	1 (1.6)	0 (0.0)	0.827	0.363	1.83	1.55–2.17
No	60 (98.4)	50 (100.0)				

^b^Adjusted for socioeconomic factor.

^*^Significant at *P* < 0.05.

95% CI = 95% confidence interval.

*N* = 111.

**Table 4:** Factors influenced the abnormality of FVC% among study respondents after controlling all the confounders.

Independent variables	*β*	S.E	*P* value	OR^b^	95% CI
Constant	1.089	0.595	0.067		
PM_2.5_	1.403	0.566	**0.013** ^*^	**4.07**	**1.34**–**12.33**
PM_10_	−0.330	0.553	0.550	0.72	0.24–2.12
VOCs	−0.056	0.517	0.913	0.95	0.34–2.60
CO_2_	−0.600	0.556	0.280	0.55	0.19–1.63
CO	2.000	0.624	**0.001** ^*^	**7.39**	**2.18**–**25.10**
Indoor smoking	−0.702	0.554	0.205	0.50	0.17–1.47
Duration living in the vicinity (year)	−3.927	0.760	<0.001^**^	0.02	0.00–0.09

^b^Adjusted for socioeconomic factor.

*N* = 111, 95% CI = 95% confidence interval, *β* = regression coefficient, and S.E = standard error.

Nagelkerke *R* square = 0.547.

^**^Significant at *P* < 0.001; ^*^significant at *P* < 0.05.

**Table 5:** Factors influenced the abnormality of FEV_1_% among study respondents after controlling all the confounders.

Independent variables	*β*	S.E	*P* value	OR^b^	95% CI
Constant	0.501	0.529	0.343		
PM_2.5_	−0.774	0.481	0.108	0.46	0.18–1.18
PM_10_	−0.233	0.505	0.645	0.80	0.30–2.13
VOCs	−0.042	0.473	0.929	0.96	0.38–2.43
CO_2_	−0.512	0.511	0.317	0.60	0.22–1.63
CO	1.648	0.545	**0.002** ^*^	**5.20**	**1.79**–**15.11**
Indoor smoking	−0.099	0.497	0.842	0.91	0.34–2.40
Years living in the vicinity	−3.217	0.681	<0.001^**^	0.04	0.01–0.15

^b^Adjusted for socioeconomic factor.

*N* = 111, 95% CI = 95% confidence interval, *β* = regression coefficient, S.E = standard error.

Nagelkerke *R* square = 0.418.

^*^Significant at *P* < 0.001; ^**^significant at *P* < 0.05.

**Table 6:** Factors influenced wheezing among study respondents after controlling all the confounders.

Independent variables	*β*	S.E	*P* value	OR^b^	95% CI
Constant	−0.654	0.376	0.082		
PM_2.5_	1.060	0.500	**0.034** ^*^	**2.89**	**1.09**–**7.68**
PM_10_	1.704	0.603	**0.005** ^*^	**5.50**	**1.69**–**17.91**
VOCs	−1.469	0.495	0.003	0.23	0.09–0.61
CO_2_	−0.584	0.455	0.200	0.56	0.23–1.36
CO	1.796	0.661	**0.007** ^*^	**6.024**	**1.65**–**22.00**
Indoor smoking	−0.102	0.479	0.831	0.90	0.35–2.31
Duration living in vicinity (year)	−0.113	0.526	0.830	0.89	0.32–2.51

^b^Adjusted for socioeconomic factor.

*N* = 111, 95% CI = 95% confidence interval, *β* = regression coefficient, S.E = standard error.

Nagelkerke *R* square = 0.134.

^*^Significant at *P* < 0.05.
